# Predictors of Long-Term Clinical Outcomes of Perceval Aortic Valve Replacements in Females: A 9-Year Experience

**DOI:** 10.3390/jcdd13100482

**Published:** 2026-09-24

**Authors:** Hiral Jhala, Charilaos Panagiotis Koutsogiannidis, Kelvin Lim, Renzo Pessotto, Vipin Zamvar, Vincenzo Giordano

**Affiliations:** Department of Cardiothoracic Surgery, Royal Infirmary of Edinburgh, Edinburgh EH16 4SA, UK

**Keywords:** sutureless, aortic valve replacement, sex differences, females, valve surgery

## Abstract

Background: Females are at increased risk of patient–prosthesis mismatch (PPM) following surgical aortic valve replacement (AVR) due to anatomical complexities. Whilst sutureless valves can reduce risk in smaller annuli, the literature lacks sex-specific outcome data pertaining to women. We evaluate long-term survival and durability outcomes pertaining to females. Methods: This is a retrospective analysis of demographic, clinical and outcome data in adults undergoing first-time isolated Perceval sutureless AVR between January 2017 and June 2025 at a tertiary centre. Subgroup analysis in females was also performed. Results: The number of patients *N* = 430 (178 males, 252 females). The median follow-up was 4.5 years. Females had higher Euroscores (*p* = 0.03) and smaller prosthetic valves (*p* = 0.01), but this group had fewer individuals with diabetes (*p* = 0.02) and fewer smokers (*p* < 0.01). Females had a higher 9-year survival (*p* = 0.02), where Euroscore (*p* = 0.02) and angina (*p* = 0.03) conferred a worse survival. No patients had PPM. FFR at 9 years was 97.3% in females (*p* = 0.61 compared to males); there were three reinterventions due to paravalvular leak. Of the females studied, 7.5% had permanent pacemakers at 9 years, with no significant predictors. At 9 years 2.4% of females had a stroke admission (mean time 1.4 ± 0.8 years), and female gender predicted stroke admission (*p* = 0.04). At 9 years, 3.1% had heart-failure admissions (mean time 1.8 ± 0.6 years), where angina (*p* = 0.02) predicted heart-failure admissions in females. Conclusion: The findings in our single-centre retrospective cohort study suggest excellent long-term survival and durability outcomes in females, however, with a greater predisposition to stroke admissions after discharge. Whilst optimization of pre-operative symptoms can potentially reduce adverse events, further larger, non-inferiority studies with echocardiographic data are required to validate our findings.

## 1. Introduction

In recent years, the adoption of sutureless aortic valve replacements (AVRs) such as the Perceval S (Sorin SGL, Saluggia, Italy)^®^ has increased significantly. With low mortality and incidence of stroke comparable to conventional sutured AVR [1], sutureless valves have also significantly reduced intraoperative times [2], serving as an excellent option for high-risk surgical patients. In addition to its self-expanding nitinol frame [3], the supra-annular anatomy of Perceval valves, with the absence of a sewing ring, serves to provide a greater indexed effective orifice area (iEOA). Consequently, patient–prosthesis mismatch (PPM), a particularly crucial problem in patients with small annuli, can be avoided. However, patients with annuli small enough to require annular enlargements will not confer the same benefit with Perceval valves [4,5]. Anatomically, females in particular, tend to have smaller, more elliptical annuli, with more complex root anatomy, narrower sinuses of Valsalva, a tighter sinotubular junction, and lower riding coronary ostia compared to their male counterparts [6,7]. Therefore, in women, there is a theoretical risk of coronary ostial damage or dynamic occlusion with tall frames, as well as paravalvular leak as a result of the ellipticity of the annulus. Furthermore, due to a smaller body surface area (BSA), prosthetic sizing is less forgiving, as there is an increased risk of PPM. In contemporary practice, Perceval valves are reserved for those patients of intermediate operative risk who do not meet the criteria for transcatheter aortic valve implantation (TAVI); however, recent European guidelines have less robust criteria pertaining to Perceval valves specifically [8]. Additionally, there is a lack of data in the literature reflecting sex-specific long-term outcomes focusing on women, whose anatomy can theoretically impact durability and post-operative complications of Perceval valves.

In our study, we evaluate the sex-specific short and long-term survival and durability outcomes in patients who have undergoing isolated Perceval aortic valve replacements at a single tertiary centre. 

## 2. Materials and Methods

### 2.1. Ethics Statement

This was a retrospective observational study with sufficient anonymization of all data; therefore, consent from individual patients and ethics review was waived.

### 2.2. Patient Recruitment

In this retrospective, single-centre observational cohort study, patients over the age of 18 years who underwent first-time isolated Perceval sutureless aortic valve replacements (AVR) between January 2017 and June 2025 were included. All data was collected contemporarily and entered onto a hospital database in a prospective manner. Out of a total of 441 patients who underwent Perceval AVR, 11 patients were excluded, as they were redo valves; 430 patients were therefore included in the final analysis (Figure 1). All patients had either a full median sternotomy or partial sternotomy, with the Perceval valve implanted in the standard fashion already described previously [9,10,11].

### 2.3. Derivation of Patient–Prosthesis Mismatch

The effective orifice area (EOA) for the Perceval valves was obtained from average EOA measurements as described previously [12]. The EOA (in cm^2^) was divided by the body surface area (BSA in m^2^) to derive the indexed effective orifice area (iEOA) as an indicator of severity of PPM. In accordance with the Valve Academic Research Consortium 3 (VARC-3) criteria for PPM [13], severe PPM was deemed at an iEOA ≤ 0.65 cm^2^/m^2^; moderate PPM was deemed at 0.65–0.85 cm^2^/m^2^ in patients with BMI < 30 kg/m^2^. For those patients with BMI ≥ 30 kg/m^2^, severe PPM was deemed at an iEOA ≤ 0.55 cm^2^/m^2^ and moderate PPM was deemed at 0.56–0.70 cm^2^/m^2^ [14].

### 2.4. Primary and Secondary Outcomes

Primary outcomes included overall survival (OS), freedom from reintervention on the aortic valve (FFR), and PPM. Secondary outcomes included major post-operative complications such as stroke (defined as any neurovascular event including transient ischaemic attacks (TIAs) or cerebrovascular attack (CVA)), permanent pacemaker insertion (PPI) and heart-failure-related readmissions after being discharged from the hospital.

### 2.5. Statistical Analysis

Demographic, clinical and intraoperative data are presented as median (interquartile range) and counts (percentage). Statistical tests were performed according to data distributions determined using the Shapiro–Wilk test. Univariate comparisons of categorical variables were performed using two-tailed Fisher’s exact tests, and continuous variables were compared using Mann–Whitney U tests. Survival and time-to-event analysis was performed using the Kaplan–Meier methods and compared using Mantel–Cox log-rank tests. Propensity score matching was abandoned to avoid considerable loss of statistical power and the risk of excluding critical clinical events. Cox proportional hazards regression was used to analyse time-to-event outcomes with no omissions to control confounding instead. Long-term outcomes are also presented as mean ± standard deviation. Proportional hazard assumptions were tested using scaled Schoenfeld residuals. These analyses were performed using GraphPad Prism 11.0.2^®^ statistical software. Statistical significance was deemed at *p* < 0.05.

## 3. Results

### 3.1. Patient Demographics and Intraoperative Data

In the total study population of 430 patients who underwent Perceval AVR, 178 (41.4%) were male and 252 (58.6%) were female. Pre-operative demographics and clinical data can be found in Table 1. Females had a higher Euroscore compared to males (*p* = 0.03); however, a greater proportion of males had a previous myocardial infarction, were smokers (both *p* < 0.01) and had diabetes (*p* = 0.02). All other pre-operative characteristics were comparable with no statistical significance. In both sexes, the majority of patients reported either no or mild symptomatology with preserved left ventricular function. Intraoperatively, males had longer cardiopulmonary bypass (*p* < 0.01) and aortic cross clamp (*p* = 0.04) times, a higher iEOA compared to females overall (*p* < 0.01), and had larger valve sizes compared to females (*p* < 0.01). 

### 3.2. Short-Term Major Post-Operative Complications

Incidences of major complications can be found in Table 2. There were no statistically significant differences between males and females for major complications. The median length of hospital stay was 7 days in both sexes, and there was no significant difference in critical care stay.

### 3.3. Survival

After a median follow-up of 4.5 years, with a complete dataset of overall survival (OS) obtained, the in-hospital mortality was 1 (0.6%) in the male population vs. 4 (1.6%) in the female population (*p* = 0.4). The survival at 1, 3 and 5 years were 88.7%, 77.9% and 63.3% in males compared to 94.0%, 88.3% and 70.5% in females (*p* = 0.02) (Figure 2). The OS at 8 years was 53.6% vs. 56.3% in males and females, respectively. In the multivariate analysis of survival, predictors of a reduced overall survival in the overall cohort were Euroscore (*p* < 0.01) and significant dyspnoea (*p* = 0.02), whereas female gender did not have any statistically significant impact on survival (Table 3). In the female cohort, Euroscore (*p* = 0.02) and significant angina (*p* = 0.03) conferred a worse survival.

### 3.4. Durability

None of the patients in the study cohort had an iEOA < 0.85 cm^2^/m^2^, meaning we had no patients with PPM. After a median follow-up of 4.5 years, five patients (1.2%) in the whole cohort had a reintervention on the aortic valve, three females (1.2%) and two males (1.1%). In the female group, two patients had a percutaneous closure of paravalvular leak, and one underwent a valve-in-valve transcatheter aortic valve implantation (TAVI). Both male patients underwent surgical redo aortic valve replacements for prosthetic valve endocarditis. The FFR at 1, 5 and 9 years was 98.8% consistently in males, compared to 99.6%, 98.8% and 97.3% in females (*p* = 0.61). In the multivariate analysis, the presence of significant angina conferred a worse FFR in the whole cohort (HR 1.4 [1.1–1.6] (*p* = 0.02); however, female gender alone, did not (Table 3). Due to small numbers of events, a multivariate analysis for predictors of FFR in the female subgroup was not performed.

### 3.5. Long-Term Complications

Long-term follow-up event data after discharge was obtained for all patients. Multivariate analysis of permanent pacemaker insertion (PPI), stroke admissions, and heart-failure admissions for the whole cohort and female subgroup can be found in Table 4. Ten males (5.6%) vs. 17 females (6.7%) underwent PPI at a later stage after discharge. The mean time to permanent pacemaker insertion was 0.4 ± 0.3 years vs. 0.8 ± 0.3 years for males and females, respectively. At 9 years, 6.1% of males and 7.5% of females had a permanent pacemaker inserted (*p* = 0.77). Female gender was not a predictor of PPI, and no statistically significant predictors were identified for the whole cohort or the female subgroup. There were no long-term stroke admissions in the male population; however, six (2.4%) females had a stroke admission, with a mean time to admission of 1.4 ± 0.79 years. At 9 years, 2.4% of females had a stroke admission. Female gender was a predictor of stroke admissions in the multivariate analysis (HR 4.1 [1.0, 7.1]; *p* = 0.04); however, in those females specifically, there were no other statistically significant predictors of stroke admissions identified. Five (2.8%) males and six (2.4%) females had a heart-failure-related admission. The mean time to heart-failure admission was 2.1 ± 1.0 years in males and 1.8 ± 0.6 years in females. At 9 years, 4.3% males and 3.1% females had a heart-failure-related admission (*p* = 0.73). Female gender was not a predictor for heart-failure admissions; however, impaired left ventricular function (HR 3.3 [0.8, 13.0]; *p* = 0.05) and pre-operative anaemia (HR 3.1 [0.9, 11.5]; (*p* = 0.05) predisposed patients to heart-failure admissions in the whole cohort. In the female subgroup, the presence of pre-operative angina (HR 10.2 [1.1, 21.6]; *p* = 0.02) and small valve size (HR 12.2 [1.4, 15.2]; *p* = 0.02) was a predictor of heart-failure admissions.

## 4. Discussion

This single-centre, retrospective, observational study aimed to explore the differences in survival, short- and long-term complications and durability after isolated Perceval AVR, in particular focusing on predictors of adverse outcomes in the female subgroup. Overall, our findings support the notion that this is a safe and successful surgical option in women. Whilst there are studies that clearly demonstrate outcomes of sutureless valves in the literature, female-specific outcomes can only be extrapolated from them to a certain extent. There is certainly a paucity in the literature of studies pertaining to sex-stratified outcomes in Perceval valves, especially of long-term data. Our study has therefore attempted to address this knowledge gap by demonstrating long-term outcomes in females and exploring predictors of those outcomes, aiming to identify potential areas for optimisation to improve outcomes in females from a real-world view.

### 4.1. Patient Characteristics and Intraoperative Findings

Overall, females had a higher risk profile compared to males, despite being of a similar age. Because of this increased risk, surgeons may be inclined to reduce intraoperative times, which can be achieved with Perceval valves [15,16], although the difference in the risk profile itself may impact long-term all-cause mortality and outcomes. Whilst more females in our cohort were anaemic, proportionally more males had a history of myocardial infarction (*p* < 0.01), pre-operative atrial fibrillation and diabetes (*p* = 0.02). Though the presence of anaemia per se was not statistically different between the sexes, it could have contributed to a larger proportion of females being more symptomatic, with 61.1% having NYHA III-IV dyspnoea compared to the males. As expected, proportionally more females had smaller-sized prostheses, as they typically have smaller annuli, making them more eligible for sutureless valves. Therefore, they had a larger representation in our study compared to males, who are more likely to receive sutured valves owing to larger annuli, which decrease the success of sutureless valve implantation [17].

### 4.2. Mortality and Short-Term Morbidity

Large multicentre, prospective studies from the CAVALIER trial [18] and Fischlein and colleagues [19] demonstrated 1-year survival rates of 96.3% with no differences or inferiority in mortality compared to sutured valves in reviews of comparative studies [20,21]. At 1 year, our females demonstrated comparable survival (94.0%) with only 1.6% in-hospital mortality. The survival advantage that our female population conferred is more apparent up to 5 years post-operatively (*p* = 0.02), after which the survivals become comparable. Whilst this data demonstrates all-cause mortality, there may be differences in cardiac-related deaths to account for this effect, especially in the context of a relatively older cohort. In the broader context of how sutureless valves fair compared to sutured AVRs, larger national cohorts such as Brown et al. [22] demonstrate a risk-adjusted survival disadvantage in females with sutured valves. The survival outcomes that we have shown in our study suggest that Perceval valves can be a good alternative option for females. In addition, due to the discrepancy between cardiac deaths and all-cause mortality, it is shown that short-term morbidity can contribute to longer-term mortality. We saw more CVAs in the females, and higher Euroscores and increasing severities of dyspnoea predicted worse survival in the whole cohort. In our female subgroup, the presence of angina was a predictor for worse overall survival. In the long term, these patients may have concomitant coronary artery disease, which may not have been severe enough to warrant surgical intervention at the time of aortic valve replacement but progressed afterwards. Therefore, it is possible that they may have undergone percutaneous revascularization or medical management, which ultimately adds to long-term morbidity and mortality. Although we excluded those patients who underwent concomitant surgical revascularization, we did not have data pertaining to the extent of co-existing coronary disease in the final group of patients. Another explanation is the rates of ventricular remodelling long-term, which differ between the sexes owing to differing aortic root geometry [7], risk of coronary obstruction [23], and coronary heights and sinus dimensions in relation to the sutureless valve frames [6].

### 4.3. Long-Term Durability and Morbidity

Our population demonstrated no PPM post-operatively, especially in females who have smaller annuli and therefore are at a theoretical risk of PPM [24]. Additionally, only 1.2% patients in the whole cohort as well as females specifically had a reintervention on the aortic valve for the duration of the study. This demonstrates promising durability, especially in females, in comparison to the benchmark set by the CAVALIER trial, which demonstrates a reintervention rate of 3.3% at 4 years [18]. The results from the CAVALIER trial included endocarditis and structural valve deterioration such as paravalvular leaks. Interestingly, out of the five patients who underwent a reintervention, the three females did not have any endocarditis and all cases were due to paravalvular leaks and therefore required percutaneous reintervention, whilst the males both had endocarditis requiring a higher-risk redo operation. It is thought from larger scale epidemiological studies and metanalyses that this is attributable to a larger prosthesis conferring a larger surface area for foreign material in males, as well as a predisposition for more staphylococcal infections causing prosthetic valve endocarditis in general [25,26]. True PPM could have been evaluated more accurately with echocardiographic data on gradients and annular dimensions should they have been available. Moreover, angina conferred a worse FFR in our cohort. This is likely due to the high burden of calcium in the aortic root, annulus and left ventricular outflow tract. Consequently, whilst these patients may have required extensive decalcification of the annulus, further decalcification may have remained incomplete to ensure adequate annular stiffness to provide sufficient anchorage and seating of the Perceval valve once deployed. It is also possible that the resultant asymmetry in these specific areas may have predisposed to paravalvular leaks in these patients; however, a high calcium burden in the native aortic annulus and root has been shown to accelerate structural stress in bioprosthetic aortic valves [27].

Both the PERSIST-AVR and CAVALIER trials consistently demonstrated high rates of PPI up to 11.8% [15,18], higher in sutureless compared to sutured valves. At 9 years, 7.5% of our females had had PPI, again comparable to the wider literature of these large- scale studies. Although there were no statistically significant predictors identified to account for this, further echocardiographic assessment of gradients, volumetric and geometric cavity measurements may have shed light on potential factors affecting PPI rates in our population. Whilst our patients did not have any pre-existing conduction dysrhythmias to warrant PPI, the depth of annular decalcification may have impacted PPI rates in females. This may in turn contribute to a higher risk of early and late post-operative stroke in females. Female gender was an independent risk factor (HR 4.1) (*p* = 0.04) for stroke admissions (3.1%) at 9 years in our cohort, which could be further evaluated with echocardiographic data looking at calcific burden, extent and distribution in relation to coronary ostia and root dimensions.

As alluded to previously, the unknown extent of coronary disease that our female patients had may have contributed to pre-operative angina, which in turn predicted heart-failure admissions in the long-term, despite lower rates compared to their male counterparts who had proportionally higher rates of previous myocardial infarctions. Overall, optimizing the symptom profile in the way of angina and breathlessness due to anaemia and impaired left ventricular function, the latter two of which predicted heart-failure admissions, could potentially reduce risk profiles and long-term outcomes.

### 4.4. Limitations

This is a retrospective, observational study performed at a single centre. There are a few limitations to be mentioned in this study. Firstly, the small sample size did not allow for comparative analysis in the form of propensity score matching whilst also retaining the power of the study. Consequently, the small number of events for long-term outcomes may have caused larger confidence intervals. Secondly, due to geographical differences in referral and follow-up pathways, we were unable to obtain sufficient echocardiographic data pre- and post-operatively to facilitate any meaningful long-term sex-stratified analysis of structural valve degeneration or ventricular remodelling. Thirdly, further imaging- based studies are required to evaluate coronary anatomy and in particular calcific extent and burden to further evaluate anatomical factors for long-term stroke, heart failure, PPI and PPM. With regards to PPM in particular, our data was based on model-specific predicted EOA as opposed to measured EOA, which can be obtained with further imaging-based studies. Nevertheless, with our data, we have demonstrated potential areas where optimization could be further evaluated, as well as demonstrating the promising long-term survival and durability benefit that Perceval AVRs confer in the female population. We hope that this serves as a starting point for further larger, comparative, non-inferiority studies comparing sex-stratified long-term outcomes.

## 5. Conclusions

In this single-centre retrospective cohort study, our findings suggest promising long-term survival and durability outcomes in females; however, there was a higher incidence of stroke admissions after discharge. Pre-operative optimization of symptoms can potentially help reduce adverse events post-operatively. However, further larger non-inferiority studies with echocardiographic data to evaluate remodelling outcomes in conjunction with clinical outcomes are required to validate our findings.

## Figures and Tables

**Figure 1:**
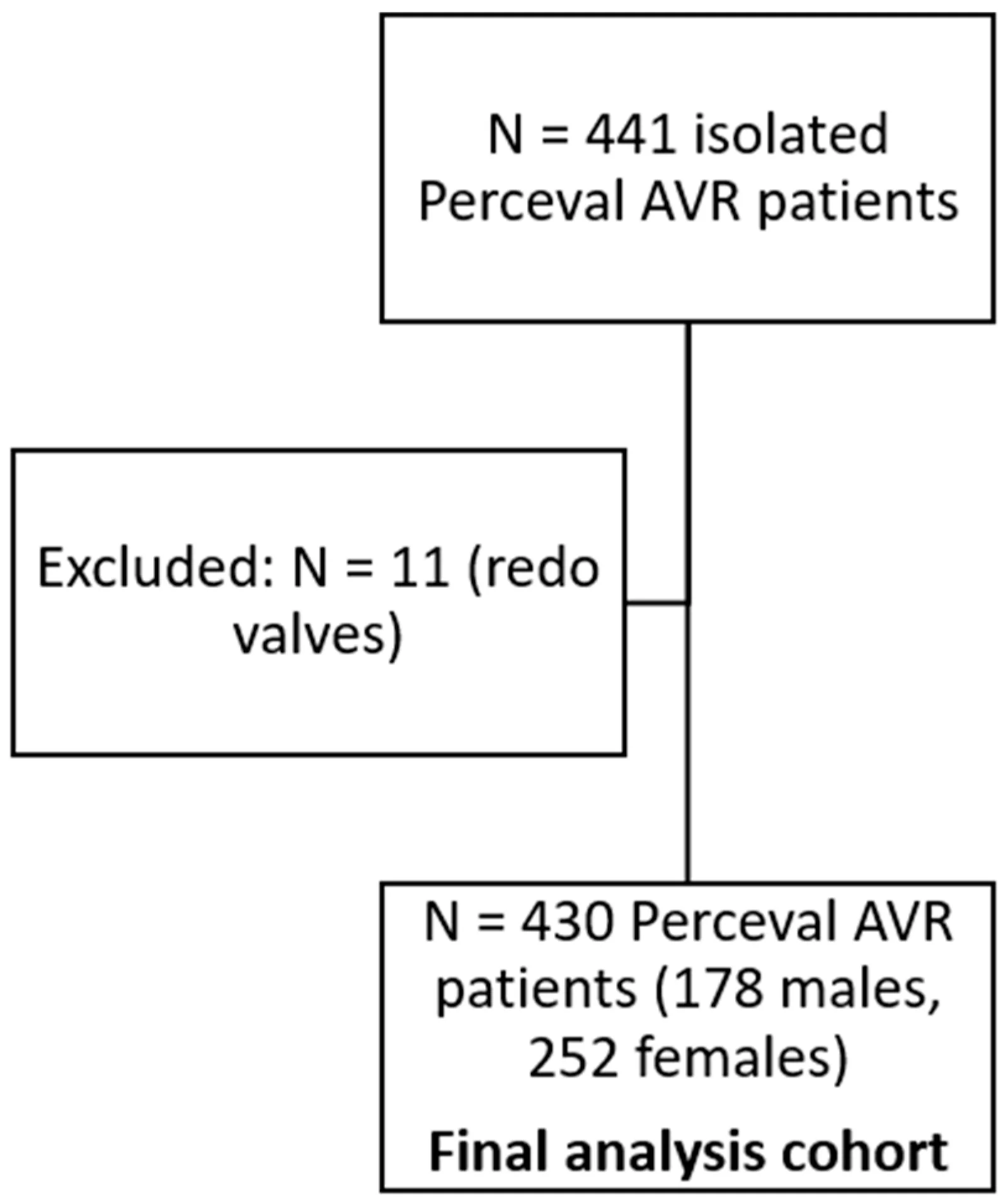
Flowchart of overall study design and exclusion criteria.

**Figure 2:**
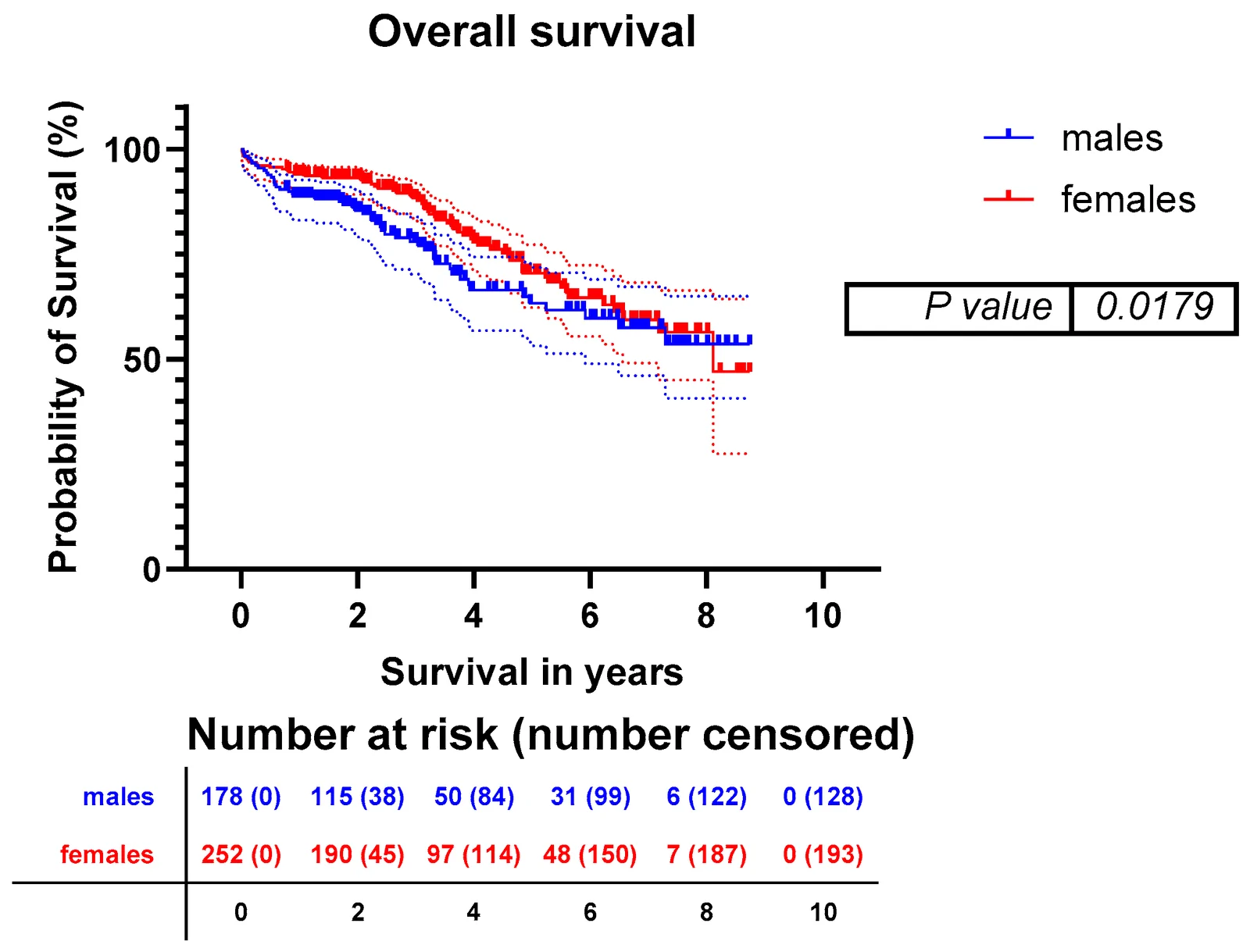
Overall survival in Perceval valves for males vs. females. 95% confidence intervals are represented by the dotted lines.

**Table 1:** Pre-operative demographics and clinical characteristics of males and females.

Characteristic	Males (*n* = 178)	Females (*n* = 252)	SMD or AR, [95% CI]	*p*-Value
Logistic euroscore (%)	2.5 (1.3, 5.5)	2.8 (1.8, 6.3)	0.2 [0.03, 0.72]	0.03
Age (years)	77.0 (74.0, 79.3)	76.0 (73.0, 79.0)	−1.0 [−1.0, 0.0]	0.33
Anaemia	63 (35.4)	85 (33.7)	1.1 [0.7, 1.6]	0.76
Urgent priority	48 (27.0)	55 (21.8)	1.3 [0.8, 2.1]	0.25
COPD	24 (13.5)	39 (15.5)	0.9 [0.5, 1.4]	0.58
Previous TIA/CVA	15 (8.4)	15 (6.0)	1.5 [0.7, 3.1]	0.34
Renal disease (dialysis)	4 (2.2)	2 (0.8)	2.9 [0.7, 15.2]	0.24
Hypertension	113 (63.5)	171 (67.9)	0.8 [0.6, 1.2]	0.35
Smoking history	110 (61.8)	117 (46.4)	1.9 [1.3, 2.7]	<0.01
Diabetes	48 (27.0)	44 (17.5)	1.7 [1.1, 2.8]	0.02
BMI (kg/m^2^)	28.1 (25.4, 31.5)	29.5 (25.4, 34.3)	1.40 [−0.16, 2.06]	0.09
Atrial fibrillation	39 (21.9)	37 (14.7)	1.6 [1.0, 2.7]	0.06
Previous MI	28 (15.7)	11 (4.4)	4.1 [1.9, 8.6]	<0.01
LVEF (%)				
>50	134 (75.3)	215 (85.3)	0.5 [0.3, 0.9]	0.01
31–50	35 (19.7)	32 (12.7)	1.7 [0.9, 2.8]	0.06
21–30	8 (4.5)	5 (2.0)	2.3 [0.7, 6.4]	0.16
<21	1 (0.6)	0 (0)	-	-
NYHA dyspnoea				
1	21 (11.8)	32 (12.7)	0.9 [0.5, 1.6]	0.88
2	70 (39.3)	66 (26.2)	1.8 [1.2, 2.8]	<0.01
3	66 (37.1)	115 (45.6)	7.1 [3.6, 14.0]	<0.01
4	21 (11.8)	39 (15.5)	0.7 [0.4, 1.3]	0.32
CCS angina				
None	91 (51.1)	139 (55.2)	0.9 [0.6, 1.3]	0.43
1	28 (15.7)	35 (13.9)	1.2 [0.7, 1.9]	0.67
2	28 (15.7)	43 (17.1)	0.9 [0.5, 1.5]	0.79
3	22 (12.4)	24 (9.5)	1.3 [0.7, 2.4]	0.35
4	9 (5.1)	11 (4.4)	1.2 [0.5, 2.8]	0.82
Intraoperative Data				
Partial sternotomy	19 (10.7)	32 (12.7)	1.2 [0.7, 2.7]	0.52
Cardiopulmonary bypass time (mins)	57.0 (47.0, 71.0)	51.0 (41.0, 66.0)	−6.0 [−9.0, −2.0]	<0.01
Cross clamp time (mins)	43.0 (34.5, 53.0)	39.0 (31.3, 50.0)	−4.0 [−6.0, 0.0]	0.04
Prosthesis size				<0.01
Small	1 (0.6)	60 (23.8)	55.3 [10.1, 563]	
Medium	22 (12.4)	120 (47.6)	0.2 [0.09, 0.3]	
Large	77 (43.3)	60 (23.8)	2.4 [1.6, 3.7]	
X-Large	78 (43.8)	12 (4.8)	15.6 [8.1, 29.8]	
iEOA (cm^2^/m^2^)	1.51 (1.37, 1.62)	1.42 (1.30, 1.55)	−0.08 [−0.1, −0.03]	<0.01

COPD = chronic obstructive pulmonary disease; TIA = transient ischaemic attack; CVA = cerebrovascular accident; BMI = body mass index; MI = myocardial infarction; LVEF = left ventricular ejection fraction; NYHA = New York Heart Association; CCS = Canadian Cardiovascular Society; iEOA = indexed effective orifice area; SMD = standardized median differences (for continuous variables); AR = attributable risk (for categorial variables); 95% CI = 95% confidence interval.

**Table 2:** Incidence of major post-operative complications in males and females.

Variable	Males	Females	SMD or AT [Females], 95% CI	*p*-Value
In-hospital mortality	1 (0.6)	4 (1.6)	0.4 [0.02, 2.1]	0.41
Permanent pacemaker	6 (3.4)	7 (2.8)	1.2 [0.4, 3.3]	0.78
Reintubation or tracheostomy	4 (2.2)	5 (2.0)	1.1 [0.3, 3.8]	>0.99
Renal replacement therapy	1 (0.6)	2 (0.8)	0.7 [0.04, 6.1]	>0.99
Cerebrovascular accident	3 (1.7)	9 (3.6)	0.5 [0.1, 1.7]	0.37
Biventricular failure	1 (0.6)	1 (0.4)	2.1 [0.1, 40.9]	0.54
Reopening for bleeding/tamponade	3 (1.7)	2 (0.8)	2.1 [0.4, 12.1]	0.65
Length of hospital stay	7.0 (5.0, 9.0)	7.0 (6.0, 11.0)	0 [0, 1.0]	0.06
Length of ICU stay	26.0 (23.0, 42.0)	27.0 (23.0, 44.0)	1.0 [−1.0, 2.0]	0.37

SMD = standard mean differences, AT = attributable risk, 95% CI = 95% confidence interval, ICU = intensive care unit.

**Table 3:** Multivariate analysis of covariates and overall survival and freedom from reintervention.

	Overall Survival		FFR		Overall Survival Females	
Covariate	Hazards Ratio (HR) [95% CI]	*p*-Value	Hazards Ratio (HR) [95% CI]	*p*-Value	Hazards Ratio (HR) [95% CI]	*p*-Value
Logistic Euroscore	1.05 [1.0, 1.1]	<0.01	0.7 [0.4, 1.0]	0.07	1.07 [1.0, 1.1]	0.02
Female	0.7 [0.45, 1.04	0.08	0.3 [0.02, 3.5]	0.35	-	-
LVEF < 50%	0.9 [0.5, 1.5]	0.71	1.0 [0.1, 2.3]	0.16	1.7 [0.9, 3.2]	0.13
NYHA III–IV	1.6 [1.1, 2.5]	0.02	5.3 [0.7, 111]	0.12	1.5 [0.8, 2.8]	0.20
CCS I–IV	1.2 [0.8, 1.8]	0.30	1.4 [1.1, 1.6]	0.02	1.8 [1.1, 3.2]	0.03
Valve size S	1.3 [0.7, 2.5]	0.37	1.9 [0.1, 44.2]	0.66	1.7 [0.8, 3.3]	0.16
iEOA (cm^2^/m^2^)	2.0 [0.6, 6.2]	0.25	0.02 [0.01, 5.8]	0.19	2.4 [0.5, 11.0]	0.29

FFR = freedom from reintervention on the aortic valve; 95% CI = 95% confidence interval; LVEF = left ventricular ejection fraction; NYHA = New York Heart Association class; CCS = Canadian Cardiovascular Society; iEOA = indexed effective orifice area.

**Table 4:** Multivariate analysis of long-term outcomes in the whole cohort and in the female subgroup.

	PPI		PPI Females		Stroke Admissions		Stroke Admissions Females		HF Admissions		HF Admissions Females	
Covariate	Hazards Ratio (HR) [95% CI]	*p*-Value	Hazards Ratio (HR) [95% CI]	*p*-Value	Hazards Ratio (HR) [95% CI]	*p*-Value	Hazards Ratio (HR) [95% CI]	*p*-Value	Hazards Ratio (HR) [95% CI]	*p*-Value	Hazards Ratio (HR) [95% CI]	*p*-Value
Female sex	1.5 [0.6, 3.6]	0.34	-	-	4.1 [1.0, 7.1]	0.04	-	-	0.4 [0.1, 1.9]	0.26	-	-
Logistic Euroscore	1.0 [0.9, 1.1]	0.68	1.0 [0.9, 1.1]	0.72	0.9 [0.7, 1.2]	0.90	1.0 [0.7, 1.2]	0.89	0.9 [0.6, 1.0]	0.11	0.9 [0.6, 1.2]	0.50
Hypertension	0.8 [0.4, 1.9]	0.66	0.5 [0.2, 1.3]	0.13	0.3 [0.1, 1.9]	0.20	0.3 [0.1, 1.9]	0.20	1.3 [0.4, 5.9]	0.71	1.4 [0.2, 13.6]	0.78
Anaemia	1.5 [0.6, 3.1]	0.35	1.4 [0.5, 3.8]	0.49	1.8 [0.3, 9.8]	0.51	1.8 [0.3, 9.8]	0.51	3.1 [0.9, 11.5]	0.05	4.0 [0.7, 10.1]	0.11
Pre-op AF	1.6 [0.6, 3.7]	0.31	2.8 [0.8, 8.4]	0.09	1.4 [0.1, 9.4]	0.80	1.4 [0.1, 9.4]	0.80	0.5 [0.02, 2.6]	0.43	1.7 [0.07, 7.1]	0.69
CCS I–IV	1.0 [0.5, 2.2]	0.99	2.1 [0.8, 6.1]	0.15	1.5 [0.2, 8.4]	0.66	1.5 [0.2, 8.4]	0.66	1.6 [0.5, 5.8]	0.44	10.2 [1.1, 21.6]	0.02
NYHA III–IV	0.7 [0.3, 1.5]	0.36	0.9 [0.3, 2.7]	0.82	1.1 [0.2, 8.6]	0.94	1.1 [0.2, 8.6]	0.94	1.3 [0.4, 5.3]	0.68	1.9 [0.3, 16.7]	0.54
LVEF < 50%	2.1 [0.8, 4.9]	0.11	1.9 [0.6, 5.6]	0.27	0.9 [0.1, 6.4]	0.90	0.9 [0.1, 6.4]	0.90	3.3 [0.8, 13.0]	0.05	1.8 [0.2, 13.2]	0.59
Valve size S	0.8 [0.2, 2.5]	0.70	0.9 [0.2, 3.0]	0.84	1.9 [0.2, 15.5]	0.53	1.9 [0.2, 15.5]	0.52	5.4 [0.8, 47.0]	0.08	12.2 [1.4, 15.2]	0.02
iEOA (cm^2^/m^2^)	0.8 [0.1, 5.8]	0.80	0.5 [0.2, 8.6]	0.64	0.03 [0.01, 9.3]	0.26	0.03 [0.01, 9.3]	0.26	0.3 [0.01, 11.0]	0.55	2.4 [0.04, 65.9]	0.78

PPI = permanent pacemaker insertion, HF = heart failure, AF = atrial fibrillation, CCS = Canadian Cardiovascular Society, NYHA = New York Heart Association, LVEF = left ventricular ejection fraction, iEOA = indexed effective orifice area.

## Data Availability

Data can be made available from the corresponding authors upon reasonable request.
